# Effects of individualized Tai-Chi on balance and lower-limb strength in older adults

**DOI:** 10.1186/s12877-019-1250-8

**Published:** 2019-08-27

**Authors:** I-Wen Penn, Wen-Hsu Sung, Chien-Hui Lin, Eric Chuang, Tien-Yow Chuang, Pei-Hsin Lin

**Affiliations:** 1 School of Medicine, Fu Jen Catholic University, New Taipei City, 24205 Taiwan; 2 Department of Physical Medicine and Rehabilitation, Fu Jen Catholic University Hospital, Fu Jen Catholic University, New Taipei City, 24352 Taiwan; 3 Department of Physical Therapy and Assistive Technology, National Yang-Ming University, Taipei, 11221 Taiwan; 4 Department of Integrative Biology Alumnus, University of California, Berkeley, CA 94720 USA; 5 Department of Physical Medicine & Rehabilitation, Taipei Veterans General Hospital and National Yang Ming University, Taipei, 11217 Taiwan; 6 Center for Rehabilitation and Technical Aids, Taipei Veterans General Hospital, Taipei, 11217 Taiwan

**Keywords:** Tai-chi, Exercise, Muscle strength, Functional balance

## Abstract

**Background:**

To investigate whether a simplified and personalized Tai-Chi program could be beneficial for practitioners. A prospective quasi-experimental observer-blinded controlled trial was done in Beitou District of Taipei City.

**Methods:**

Community-dwelling adults aged 65 and older without debilitating disease (*N* = 50) participated the study. Those who were willing to participate in exercise program were assigned to individualized Tai-Chi (iTC) group (*n* = 20), receiving iTC training for 8 weeks, and traditional Tai-Chi (tTC) group (*n* = 15), receiving tTC training for 8 weeks. Those who were not willing to participate in exercise training were included in the control group (*n* = 15). Functional balance tests, the Berg Balance Scale (BBS), timed up-and-go (TUG) test, functional-reach test, and measurement of lower-extremity muscle strength were conducted before and 8 weeks after the intervention.

**Results:**

Significant improvements were noted in all functional balance tests and strength assessments of 16 major lower-limb muscle groups in participants of the iTC group compared to the control group, whereas only BBS and muscle strength of hips and ankles were improved in the tTC group. Practitioners of iTC outperformed tTC in BBS and strength of two major muscles.

**Conclusions:**

Personalized Tai-Chi training designed based on an objective measurement and conducted according to graded intensity and complexity benefitted practitioners after a short period.

**Trial registration:**

Trial registration number: ClinicalTrials.gov ID: NCT03659396, Unique Protocol ID: 1000087

Date of registration: 03/28/2017

The trial was registered retrospectively

## Background

Tai-Chi, characterized by a sequence of gentle, low-impact, and coordinated movements, is an appropriate form of exercise for older adults [1] because it involves minimal strain on joints and the cardiovascular system. In addition to the benefits from general physical activity, such as improving cardiopulmonary fitness and lowering blood pressure [2–5], Tai-Chi increases muscle strength in the lower extremities, improves balance control, proprioception, and postural adaption, and reduces the risk of falling in older adults [6–9].

Individuals of a similar biological age may exhibit a wide variance in their responses to exercise because of varying aging processes [1]. Thus, the prescription of Tai-Chi exercise should be personalized with regard to the intensity and difficulty, and exercise levels should gradually progress based on the functional ability of practitioners [10]. Moreover, the complexity and duration of traditional Tai-Chi (tTC) sequences may be too challenging for some older adults to execute [11, 12]. Therefore, we hypothesize that compared with a tTC program, an individualized Tai-Chi (iTC) program may be more effective in achieving the exercise’s training goals, maximizing beneficial effects, and improving exercise adherence.

Several simplified Tai-Chi programs have been developed [11–14]. However, these exercises have primarily been designed in accordance with experts’ opinions without considering participants’ ability, and the training sessions have not involved graded progression because participants performed exercises at the same level of intensity and complexity throughout the program. We previously reported the development of a personalized Tai-Chi exercise program for older adults based on the measurements of practitioners’ center of pressure displacement [15]. However, few participants were included, only the strength of the knee extensor was assessed, and a control group was not included; nevertheless, the findings of that study formed the basis of the present study for prescribing iTC moves according to participants’ balance abilities and executing exercises in a graded manner as practitioners improved their balance control [15, 16]. In the present study, we investigated the effect of an iTC regimen on functional balance control and lower-extremity muscle strength and compared the results of the iTC exercise group with those of control and tTC exercise groups.

## Methods

This study was a prospective quasi-experimental observer-blinded controlled trial. Participants received baseline assessments and were followed up after 8 weeks. The study was approved by the Institutional Review Board of National Yang-Ming University #1000087. Written informed consent was obtained from all participants.

### Participants

**Fig. 1:**
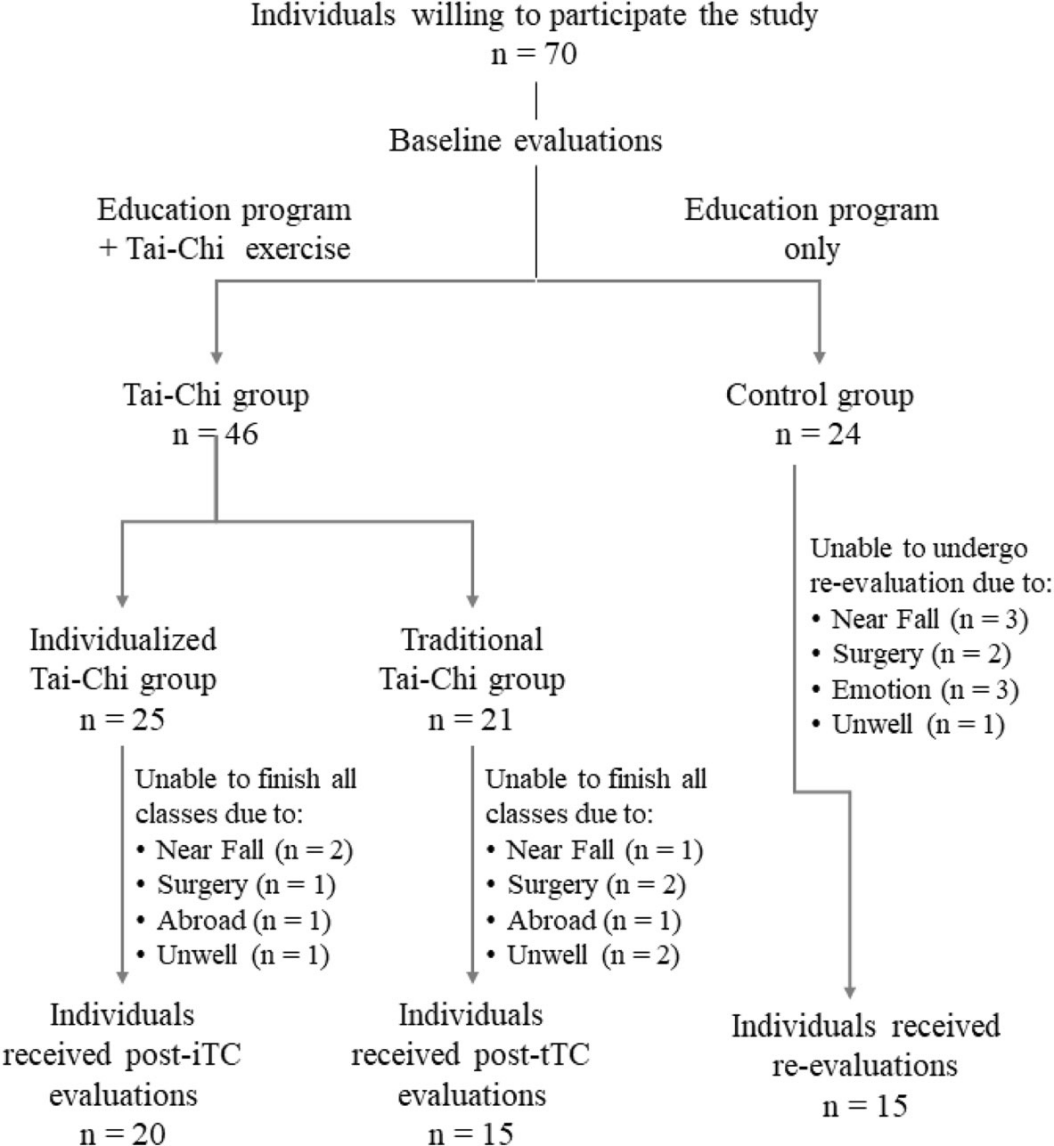
Flowchart explaining assignment of the participants to the study and control groups

This is a quasi-experimental observer-blinded controlled trial study. Participants were recruited in Beitou District of Taipei City. As shown in Fig. 1, 70 individuals were initially recruited to the study. Those who were unwilling to practice Tai-Chi were assigned to the control group (*n* = 24), while those joined the Tai-Chi group were free to choose between joining either iTC (*n* = 25) or tTC (*n* = 21) group to enhance their stays to the program. All participants received education program that contained health information related to exercise, nutrition, fall, and balance. Participants of the iTC and tTC groups were asked to attend 24 Tai-Chi exercise classes, 3 classes per week, 30 min per class for 8 weeks. Some of them, however, failed to complete all 24 classes due to the reasons listed in Fig. 1; their data were not included in the analyses. The post-intervention evaluation was performed in 20, 15, and 15 individuals of the iTC, tTC, and control group, respectively (Fig. 1).

### Inclusion criteria

The inclusion criteria for the study were as follows: (a) aged 65 or older; (b) can understand and follow simple verbal instructions to perform exercises; (c) can stand independently; (d) can walk independently (with or without assistive devices); and (e) can stand up from a chair independently (with or without assistive devices). The evaluation was performed by a certified physical therapist.

### Exclusion criteria

The exclusion criteria were as follows: (a) presence of any disorders of the central nervous system; (b) any orthopedic or cardiovascular disorders impeding ambulatory function or the ability to stand; (c) severe hearing impairment that inhibits ability to follow evaluation instructions and therapist guidance; (d) severe visual impairment that influences the individual’s safety during execution of exercises; (e) participation in other similar trials; (f) any lower-limb fractures in the preceding 6 months; and (g) prior experience in Tai-Chi exercise for more than 2 months.

### Assignment and blinding

Community-dwelling individuals who joined the study but were unwilling to practice Tai-Chi was recruited to the control group and received only the education program. Those who were willing to perform Tai-Chi exercises in addition to the education program could choose to join either iTC exercise or tTC exercise group. The current study is a forerunner to a randomized controlled trial. The pre- and post-intervention evaluations were performed by physical therapists who were blinded to the exercise treatments.

### Individualized tai-chi exercise group

We first evaluated participants’ COP displacement by using a computerized evaluation system that consisted of a computer, a force platform measuring an area of 100 × 100 cm2, and strain gauges attached to load cells located at the four corners of the force plate. Strain gauges acquired a signal input for COP displacement. The classical 24-form Yang-style Tai-Chi exercise were classified into sixteen foot-support patterns, including four spatial directions (anterior, posterior, right, and left) at four feet positions (parallel stance, standing with the feet shoulder-width apart, left foot forward, and right foot forward), and four difficulty levels. At each feet-position, participants were asked to lean forward, backward, to the left, and to the right as far as possible at a comfortable, self-selected pace and maintain balance for 5 s. We divided all Tai-Chi moves into four difficulty levels by using normalized COP displacement at anteroposterior and mediolateral directions: moves with COP displacement < 25, 25–50%, 50–75%, and > 75% of normalized values were classified as level 1, 2, 3 and 4, respectively [15]. Based on the results of the maximum COP displacement and individual endurance, participants were assigned 3–5 moves at an appropriate difficulty level in each session. Participants repeated these selected moves for 30 min in each training session, three sessions per week for 8 weeks. The exercise program commenced with a low-difficulty level move and then progressed to higher difficulty levels as participants became adept at each move [17].

### Traditional tai-chi exercise group

Participants in this program learned and practiced a classical 24-form Yang-style Tai-Chi exercise [18] under the instruction of certified Tai-Chi masters. The exercise program consisted of three sessions per week for 8 weeks. The duration of each session was 30 min. For each session, participants usually practiced a complete Yang-Style Tai-Chi exercise two or three times.

### The control group

Participants who were unwilling to participate in the exercise program were included as the control group. After the baseline assessments, participants attended education classes and received information related to nutrition, exercise, fall, and balance. The follow-up assessments were performed 8 weeks later; those failed to attend such evaluations due to the reasons listed in Fig. 1 were excluded from the study.

### Outcome measurements

Functional balance tests, including Berg Balance Scale (BBS), timed up-and-go (TUG) test, functional-reach test, and measurement of lower-extremity muscle strength were conducted before and 8 weeks after the intervention by a certified physical therapist who was blinded to the treatment allocation. The BBS test consisted of 14 items that evaluated individuals’ balance in sitting, standing, and changing positions. Scoring was determined using an ordinal 5-point scale with a total score range from 0 to 56, where higher scores indicated superior balance [19].

The TUG test is a simple test used to assess a person’s mobility and requires both static and dynamic balance [20]. The test measures the time taken by participants to stand up from a chair, walk 3 m at a comfortable speed, turn around, return to the chair, and then sit down. Three timed trials are needed to ensure performance stability in the TUG test. Therefore, mean values for three trials, with 1-min rest between each trial, were used for analysis.

The functional-reach test was used to assess dynamic balance; this test measured the maximal distance participants could reach forward beyond the length of their own arms. Participants were asked to stand next to a wall and reach forward as far as possible without moving their feet. The additional reaching distance was then recorded in centimeters [21].

Lower-extremity muscle strength was measured using a hand-held isometric dynamometer (Micro FET®3, Hoggan Health Industries). The portable digital dynamometer performs accurate, objective muscle testing and enables assessors to stabilize and assist individuals while keeping one hand free. In this study, the following lower-limb muscles were bilaterally measured: hip flexor, hip extensor, hip abductor, hip adductor, knee extensor, knee flexor, ankle dorsiflexor, and ankle plantar flexor. The participants were asked to perform maximal isometric contractions for at least 3 s.

### Statistical analysis

A one-way ANOVA was conducted to compare the three groups with respect to their baseline characteristics, such as age, weight, height, and lower-limb muscle strength. Paired t-tests were conducted to compare the baseline and follow-up assessments in each group. A two-way repeated measures ANOVA was conducted to test the effect of interaction between time and group to compare the baseline and follow-up assessment of forward-reach distance, up-and-go time, and lower-limb muscle strength. To further explain the interaction, the simple effect of time at each group was also tested. All statistical analyses were performed using STATA version 14.2 (College Station, TX). Statistical significance was defined as a *p* value of < 0.05.

## Results

### Demographic characteristics of participants in the three groups

The sample size determined using G*Power 3.1.9.4 software was based on the results of our pilot study (Sung et al., 2018). With 80% power assuming an effect size of 0.5 at a two-tailed significance level of 0.05, the calculated sample size was at least 14 participants in each group. We initially recruited 70 participants, of whom 46 received the education program along with Tai-Chi courses and 24 received only the education program. The flowchart of the study is presented in the additional file (Fig. 1). Participants of both Tai-Chi groups were required to complete all 24 exercise sessions (30 min each), which were performed three times a week for 8 weeks. All classes were supervised by the study controllers and at least one of the five Tai-Chi masters. Of the 25, 21, and 24 participants assigned to iTC, tTC, and control groups, 5, 6, and 9 individuals could not finish the program, respectively. Finally, a total of 50 participants underwent the post-intervention evaluation. The demographic characteristics of participants who completed the study are listed in Table 1. No significant difference was observed in the distribution of age, sex, height, and weight among the iTC, tTC, and control groups.

**Table 1:** Demographic characteristics of participants

	Individualized Tai-Chi Group (*n* = 20)	Traditional Tai-Chi Group (*n* = 15)	Control Group (*n* = 15)	*p* value
Age (y)	76.45 ± 8.63	75.27 ± 5.20	73.4 ± 8.2	0.515
Sex				0.872
Male	2 (10%)	2 (13.3%)	3 (20%)	
Female	18 (90%)	13 (86.6%)	12 (80%)	
Ht (cm)	153.57 ± 7.88	155.8 ± 6.6	154.67 ± 6.66	0.667
Wt (kg)	53.70 ± 8.30	57.9 ± 7.87	52.73 ± 6.24	0.125

*Abbreviations*: *y* years, *Ht* Height, *Wt* Weight

### No significant difference observed between the baseline assessment results of the three groups

Table 2 presents the results of participants in the iTC, tTC, and control group for the baseline assessments, which consisted of three functional balance tests and a lower-limb muscle strength assessment. No significant difference was observed among the three groups in the functional balance assessments. For muscle-strength measurements, the tTC group only exhibited higher scores for the right hip abductor (*p* = 0.017) and right knee flexor (*p* = 0.025) compared with iTC and control groups.

**Table 2:** Baseline assessments of participants

	Individualized Tai-Chi Group (*n* = 20)	Complete Tai-Chi Group (*n* = 15)	Control Group (*n* = 15)	*p* value
Functional Balance Assessment				
BBS	45.05 ± 12.17	49.33 ± 4.52	45.13 ± 12.35	0.944
TUG (seconds)	12.00 ± 8.45	9.10 ± 1.94	11.24 ± 9.59	0.527
FRD (cm)	24.30 ± 7.40	23.75 ± 6.47	21.66 ± 7.01	0.531
Muscle Strength				
RH F	14.55 ± 3.71	16.39 ± 4.32	13.64 ± 5.87	0.261
LH F	14.47 ± 3.32	15.29 ± 4.72	13.09 ± 5.00	0.373
RH E	13.64 ± 4.91	16.06 ± 3.33	12.67 ± 5.37	0.129
LH E	14.36 ± 5.49	15.71 ± 3.46	12.57 ± 4.58	0.194
RH Ab	12.67 ± 3.66	15.51 ± 3.73	12.04 ± 2.65	0.016*
LH Ab	12.57 ± 3.20	12.26 ± 2.07	12.31 ± 3.08	0.942
RH Ad	10.23 ± 2.86	11.07 ± 2.09	9.38 ± 3.02	0.240
LH Ad	9.67 ± 2.63	11.88 ± 2.91	9.74 ± 3.12	0.057
RK F	11.20 ± 2.83	13.13 ± 2.84	10.09 ± 3.31	0.025*
LK F	10.64 ± 2.92	12.81 ± 2.69	10.16 ± 3.99	0.061
RK E	15.93 ± 4.89	18.06 ± 3.92	15.15 ± 6.48	0.286
LK E	15.58 ± 5.46	16.91 ± 3.08	13.95 ± 5.06	0.240
RA Df	11.10 ± 2.85	11.87 ± 3.01	10.57 ± 2.80	0.466
LA Df	11.32 ± 2.65	12.01 ± 2.81	10.85 ± 2.43	0.484
RA Pf	15.41 ± 3.71	17.12 ± 2.46	16.06 ± 5.10	0.441
LA Pf	14.88 ± 3.96	16.53 ± 2.90	15.51 ± 4.85	0.484

*Abbreviations*: *BBS* Berg Balance Scale, *TUG* Timed up-and-go, *FRD* Forward reach distance, *RH* Righ hip, *F* Flexor, *LH* Left hip, *E* Extensor, *Ab* Abductor, *Ad* Adductor, *RK* Right knee, *LK* Left knee, RA Right ankle, *Df* Dorsi-flexor, *LA* Left ankle, *Pf* Plantar-flexor

*p < 0.05

### Functional balance and muscle strength improved after tai-chi interventions

As shown in Table 3, participants in the iTC group exhibited a significant improvement in three types of functional balance tests and all 16 groups of lower-limb muscle strength (*p* < 0.001–0.007). In the tTC group, only the BBS score (*p* = 0.005) and muscle strength of the bilateral hip flexors, right hip extensor, left hip abductor, right hip adductor, and bilateral ankle dorsiflexor improved significantly (*p* = 0.010–0.033). The control group also exhibited improved muscle strength of the right hip extensor (*p* = 0.033). The control group received health information regarding exercises and prevention of falls and underwent the same functional balance tests as did the other groups; they were also informed about the test results. This education program might have increased their awareness of exercise and encouraged self-training.

**Table 3:** Baseline and follow-up assessment results of participants

Individualized Tai-Chi Group (*n* = 20)				Traditional Tai-Chi Group (*n* = 15)			Control Group (*n* = 15)		
	Baseline	Follow-up	*p* value	Baseline	Follow-up	*p* value	Baseline	Follow up	*p* value
Functional Balance									
BBS	45.05 ± 12.17	48.00 ± 11.35	0.000*	49.33 ± 4.52	51.33 ± 4.79	0.000*	45.13 ± 12.35	45.87 ± 12.36	0.098
TUG	12.00 ± 8.45	10.10 ± 7.65	0.000*	9.10 ± 1.94	8.33 ± 1.71	0.050	11.24 ± 9.59	10.52 ± 8.86	0.075
FRD	24.30 ± 7.40	26.24 ± 6.82	0.005*	23.75 ± 6.47	25.52 ± 4.11	0.299	21.66 ± 7.01	22.03 ± 6.65	0.728
Muscle Strength									
RH F	14.55 ± 3.71	17.71 ± 3.84	0.002*	16.39 ± 4.32	18.88 ± 3.25	0.011*	13.64 ± 5.87	14.48 ± 4.57	0.340
LH F	14.47 ± 3.32	16.98 ± 3.86	0.005*	15.29 ± 4.72	17.90 ± 3.75	0.010*	13.09 ± 5.00	13.89 ± 4.45	0.393
RH E	13.64 ± 4.91	17.31 ± 5.60	0.000*	16.06 ± 3.33	18.10 ± 3.85	0.026*	12.67 ± 5.37	14.65 ± 5.00	0.033*
LH E	14.36 ± 5.49	16.98 ± 3.86	0.001*	15.71 ± 3.46	17.90 ± 3.75	0.083	12.57 ± 4.58	13.89 ± 4.45	0.128
RH Ab	12.67 ± 3.66	15.52 ± 4.72	0.004*	15.51 ± 3.73	16.94 ± 2.54	0.112	12.04 ± 2.65	13.09 ± 2.77	0.088
LH Ab	12.57 ± 3.20	14.90 ± 4.21	0.007*	12.26 ± 2.07	16.00 ± 2.87	0.000*	12.31 ± 3.08	12.01 ± 2.54	0.623
RH Ad	10.23 ± 2.86	13.06 ± 4.20	0.002*	11.07 ± 2.09	12.92 ± 2.99	0.033*	9.38 ± 3.02	9.61 ± 3.21	0.638
LH Ad	9.67 ± 2.63	12.58 ± 3.59	0.000*	11.88 ± 2.91	13.53 ± 2.68	0.054	9.74 ± 3.12	10.20 ± 2.36	0.308
RK F	11.20 ± 2.83	13.86 ± 3.87	0.000*	13.13 ± 2.84	14.52 ± 2.70	0.096	10.09 ± 3.31	10.61 ± 2.73	0.367
LK F	10.64 ± 2.92	13.53 ± 4.59	0.001*	12.81 ± 2.69	14.15 ± 2.94	0.090	10.16 ± 3.99	10.73 ± 2.85	0.435
RK E	15.93 ± 4.89	20.47 ± 5.81	0.000*	18.06 ± 3.92	19.06 ± 4.40	0.220	15.15 ± 6.48	15.24 ± 4.77	0.936
LK E	15.58 ± 5.46	18.19 ± 6.11	0.007*	16.91 ± 3.08	18.21 ± 3.66	0.154	13.95 ± 5.06	14.20 ± 3.82	0.793
RA Df	11.10 ± 2.85	13.88 ± 3.43	0.001*	11.87 ± 3.01	14.00 ± 2.80	0.010*	10.57 ± 2.80	11.81 ± 2.69	0.140
LA Df	11.32 ± 2.65	14.26 ± 3.40	0.000*	12.01 ± 2.81	14.47 ± 3.18	0.003*	10.85 ± 2.43	11.52 ± 2.78	0.263
RA Pf	15.41 ± 3.71	20.46 ± 6.74	0.000*	17.12 ± 2.46	19.09 ± 3.66	0.068	16.06 ± 5.10	17.09 ± 4.64	0.280
LA Pf	14.88 ± 3.96	20.27 ± 6.53	0.000*	16.53 ± 2.90	17.44 ± 3.90	0.220	15.51 ± 4.85	15.57 ± 4.61	0.936

*Abbreviations*: *BBS* Berg Balance Scale, *TUG* Timed up-and-go, *FRD* Forward reach distance, *RH* Righ hip, *F* Flexor, *LH* Left hip, *E* Extensor, *Ab* Abductor, *Ad* Adductor, *RK* Right knee, *LK* Left knee, *RA* Right ankle, *Df* Dorsi-flexor, *LA* Left ankle, *Pf* Plantar-flexor

*p < 0.05

### Participants benefitted more from individualized tai-chi than from traditional tai-chi

Table 4 presents the comparison of the results for the three groups based on 2-way repeated measures ANOVA. The interaction between time and group showed statistical significance over BBS, muscle strength over left hip abductor, right hip adductor, left hip adductor, right knee extensor, left ankle dorsi-flexor, right ankle plantar flexor and left ankle plantar flexor. Further analysis of the simple effect of time at each group showed no significant difference between baseline and follow-up assessment of all the measurements in the control group. By contrast, in the iTC group, there were significant differences between baseline and follow-up assessments of all the measurements except forward-reach distance. In the tTC group, some of the assessments showed significant differences between baseline and follow-up assessment. They were BBS, muscle strength over right hip flexor, right hip extensor, left hip abductor, right hip adductor, left hip adductor, right knee flexor, right ankle dorsi-flexor and left ankle dorsi-flexor.

**Table 4:** Comparisons among Individualized Tai-Chi, Traditional Tia-Chi and Control group using Two-way Repeated Measures ANOVA

	*p* values for Repeated Measures ANOVA			*p* values for testing the simple effect of time on Group		
	Group	Time	The Interaction between Time and Group	Individualized Tai-Chi Group	Traditional Tai Chi Group	Control Group
Functional Balance Assessment						
BBS	0.4033	0.0000*	0.0019*	0.000*	0.000*	0.104
TUG	0.5991	0.0000*	0.0520	0.000*	0.069	0.087
FRD	0.2537	0.0376*	0.5537	0.058	0.130	0.749
Muscle strength						
RH F	0.0500	0.0001*	0.1677	0.000*	0.010*	0.370
LH F	0.0757	0.0002*	0.2739	0.002*	0.006	0.376
RH E	0.1295	0.0000*	0.1999	0.000*	0.016*	0.018*
LH E	0.1215	0.0001*	0.0736	0.000*	0.139	0.146
RH Ab	0.0091	0.0004*	0.2362	0.000*	0.098	0.221
LH Ab	0.1291	0.0000*	0.0017*	0.001*	0.000*	0.696
RH Ad	0.0358*	0.0003*	0.0442*	0.000*	0.019*	0.759
LH Ad	0.0227*	0.0000*	0.0309*	0.000*	0.019*	0.493
RK F	0.0058*	0.0001*	0.0538	0.000*	0.039*	0.427
LK F	0.0363*	0.0004*	0.0698	0.000*	0.088	0.458
RK E	0.1118	0.0008*	0.0017*	0.000*	0.296	0.930
LK E	0.0794	0.0110*	0.1773	0.003*	0.177	0.790
RA Df	0.1552	0.0000*	0.3439	0.000*	0.010*	0.121
LA Df	0.0799	0.0000*	0.0439*	0.000*	0.001*	0.337
RA Pf	0.5485	0.0000*	0.0114*	0.000*	0.063	0.325
LA Pf	0.3887	0.0001*	0.0001*	0.000*	0.325	0.943

*Abbreviations*: *BBS* Berg Balance Scale, *TUG* Timed up-and-go, *FRD* Forward reach distance, *RH* Righ hip, *F* Flexor, *LH* Left hip, *E* Extensor, *Ab* Abductor, *Ad* Adductor, *RK* Right knee, *LK* Left knee, *RA* Right ankle, *Df* Dorsi-flexor, *LA* Left ankle, *Pf* Plantar-flexor

*p < 0.05

## Discussion

Tai-Chi is based on continuous fluid movements involving a semi-squat posture that enables an individual’s bodyweight to be shifted from side to side [22, 23]. Concentric and eccentric muscle contractions performed in Tai-Chi can increase lower-limb muscle strength [7, 22]. One previous study focused on the beneficial effect of Tai-Chi training on the muscle strength of the knee extensors or flexors in older adults [24], whereas other studies have reported the effects of Tai-Chi training on the hip and ankle muscles [25–27]. Thus, a range of results have been reported due to the employment of different Tai-Chi moves, training durations, and assessment methods [28].

In this study, we found that although both iTC and tTC training were beneficial for practitioners compared with controls, the 8-week iTC training could improve balance and muscle strength of all lower limbs, whereas tTC appeared to improve only the score of the BBS and the strength of some hip and ankle muscles but not knee flexors and extensors, which is in contrast to the findings of some previous studies [8, 29–31]. This result may indicate one of the following. (a) The intervention duration was too short for each Tai-Chi move to affect the knee muscles. Additionally, training was affected by the semi-squat posture of Tai-Chi, which induces stress on the knee muscles [23, 24]. (b) The full set of tTC exercises may have involved moves that were too difficult for the elderly participants. The amplitude of weight shifting, as estimated by the degree of COP displacements, differs among various Tai-Chi moves [32]. Individuals vary in their abilities to voluntarily shift their weight in various spatial directions and to briefly maintain stability in different positions; thus, not all older individuals are able to complete Tai-Chi programs. In this study, we noted that participants in the tTC group often omitted or skipped difficult moves or performed the steps at their own will. Therefore, elderly participants may require individual consideration when a Tai-Chi exercise program is being developed for them [33]. The present study revealed that the beneficial effects associated with iTC exercises were superior to those observed from a tTC program. The participants of the iTC group in this study were asked to repeatedly practice only 3–5 moves in each session; this format enabled them to meet the trainer’s requirements and experience the beneficial effects of the training sequence. The moves of iTC program were assigned according to the individual’s balance control, as assessed through COP measurements. The training program was designed to be progressive and continually challenge participants. This goal was achieved by gradually incorporating Tai-Chi moves of increasingly substantial COP displacements [34, 35]. After the initial moves had been learned and mastered, the difficulty of the exercise routine was increased to the next level in a graded manner. We therefore concluded that the superior results observed in the iTC group may have been attributable to the tailored moves and graded intensity and complexity mode of practices.

Our results agree with those of other studies that have reported significant improvements in leg balance from Tai-Chi exercises. However, these results contradict those reported in studies conducted by Woo et al. [36], which indicated that Tai-Chi training alone is not sufficient for improving balance. This divergence may be a result of the intensity and duration of the Tai-Chi intervention as well as the fact that muscle strength is only one factor that influences balance. Most studies have reported that older adults often must practice for relatively long periods, typically from 12 to 48 weeks, to benefit from various forms of exercise, including Tai-Chi [8, 29–31]. Notably, a mere 8-week intervention as described in the present study, provided similar beneficial effects as those reported in longer interventions. If the participants begin training at an intensity and complexity suited to them then they can more precisely perform sequences designed to improve muscle strength, neuromuscular control, proprioception, postural adaption, and balance [8, 25]. Although balance abilities associated with the knee extensor and flexor are primary contributors to balance control, our results did not indicate a relationship between the knee extensor and flexor and improvements in BBS in tTC. Tai-Chi is regarded as an effective means of enhancing collective motor functions; the movements stimulate part of the brain that governs balance, thereby increasing overall steadiness [37–39].

The primary limitation of the present study was its quasi-experimental design. The findings of our exploratory analysis revealed that participants who were unwilling to join the exercise group tended to be older than participants who were willing to join the exercise group. Furthermore, the single-blind design used in this study may have caused a placebo effect, and the study involved only individual measurements of muscle power of each lower limb instead of a summary measure.

## Conclusions

We found that the personalized Tai-Chi training program designed on the basis of an objective measurement and conducted according to graded intensity and complexity was more beneficial to practitioners after a short period of 8 weeks compared with the traditional full-course Tai-Chi training program.

## Data Availability

Requests for data and associated analyses with individuals’ IDs removed or appropriately concealed can be addressed to Dr. Wen-Hsu Sung of NYMU.

## Abbreviations

BBS: Berg Balance Scale
COP: Center of pressure
Ht: Height
iTC: individualized Tai-Chi
LOS: Limit of stability
tTC: traditional Tai-Chi
TUG: Timed up-and-go
Wt: Weight
y: year
